# Delivery Mode and the Transition of Pioneering Gut-Microbiota Structure, Composition and Predicted Metabolic Function

**DOI:** 10.3390/genes8120364

**Published:** 2017-12-04

**Authors:** Noel T. Mueller, Hakdong Shin, Aline Pizoni, Isabel C. Werlang, Ursula Matte, Marcelo Z. Goldani, Helena A. S. Goldani, Maria G. Dominguez-Bello

**Affiliations:** 1 Department of Epidemiology, Johns Hopkins Bloomberg School of Public Health, Baltimore, MD 21205, USA; 2 Welch Center for Prevention, Epidemiology and Clinical Research, Baltimore, MD 21205, USA; 3 Department of Food Science and Biotechnology, College of Life Science, Sejong University, Seoul 05006, Korea; hakdong.shin@gmail.com; 4 Post Graduate Program Sciences in Gastroenterology and Hepatology, Federal University of Rio Grande do Sul, Porto Alegre, RS 90040-060, Brazil; pizoni.aline@gmail.com (A.P.); hgoldani@hcpa.edu.br (H.A.S.G.); 5 Post Graduate Program in Child and Adolescent Health, Federal University of Rio Grande do Sul, Porto Alegre, RS 90040-060, Brazil; iwerlang@yahoo.com.br (I.C.W.); umatte@hcpa.edu.br (U.M.); mgoldani@hcpa.edu.br (M.Z.G.); 6 Division of Translational Medicine, New York University School of Medicine, New York, NY 10016, USA

**Keywords:** cesarean section, microbiome, microbiota, microbial community, obesity

## Abstract

Cesarean (C-section) delivery, recently shown to cause excess weight gain in mice, perturbs human neonatal gut microbiota development due to the lack of natural mother-to-newborn transfer of microbes. Neonates excrete first the in-utero intestinal content (referred to as meconium) hours after birth, followed by intestinal contents reflective of extra-uterine exposure (referred to as transition stool) 2 to 3 days after birth. It is not clear when the effect of C-section on the neonatal gut microbiota emerges. We examined bacterial DNA in carefully-collected meconium, and the subsequent transitional stool, from 59 neonates [13 born by scheduled C-section and 46 born by vaginal delivery] in a private hospital in Brazil. Bacterial DNA was extracted, and the V4 region of the 16S *rRNA* gene was sequenced using the Illumina MiSeq (San Diego, CA, USA) platform. We found evidence of bacterial DNA in the majority of meconium samples in our study. The bacterial DNA structure (i.e., beta diversity) of meconium differed significantly from that of the transitional stool microbiota. There was a significant reduction in bacterial alpha diversity (e.g., number of observed bacterial species) and change in bacterial composition (e.g., reduced Proteobacteria) in the transition from meconium to stool. However, changes in predicted microbiota metabolic function from meconium to transitional stool were only observed in vaginally-delivered neonates. Within sample comparisons showed that delivery mode was significantly associated with bacterial structure, composition and predicted microbiota metabolic function in transitional-stool samples, but not in meconium samples. Specifically, compared to vaginally delivered neonates, the transitional stool of C-section delivered neonates had lower proportions of the genera *Bacteroides*, *Parabacteroides* and *Clostridium*. These differences led to C-section neonates having lower predicted abundance of microbial genes related to metabolism of amino and nucleotide sugars, and higher abundance of genes related to fatty-acid metabolism, amino-acid degradation and xenobiotics biodegradation. In summary, microbiota diversity was reduced in the transition from meconium to stool, and the association of delivery mode with microbiota structure, composition and predicted metabolic function was not observed until the passing of the transitional stool after meconium.

## 1. Introduction

C-section delivery is a common surgical procedure intended to increase the chances of successful delivery and to protect the health of the mother. Yet this highly overused intervention [1,2,3,4] has been associated with higher offspring risk of immune and metabolic disorders [5,6,7,8] and shown to cause excess weight gain in a murine model [9]. It is hypothesized that these associations are due to C-section-delivered neonates not receiving the full inoculum of maternal microbes at birth [7].

Neonates delivered vaginally are exposed to maternal vaginal microbiota in labor, and additionally to maternal intestinal microbiota at birth [10,11,12,13]. C-section and the prophylactic antibiotics administered during this surgery [14] may cause newborns to miss this natural microbial inoculum [14,15,16]. C-section-delivered neonates are first exposed to microbes from the delivery room [17] and maternal skin (e.g., *Staphylococcus* and *Corynebacterium*) [15]. Recent longitudinal studies suggest the postnatal impact of delivery mode on the infant microbiome may extend for up to two years [16,18]. Yet, since the intestine of the newborn at birth is filled with in-utero contents, which are passed postnatally as meconium, it is unclear when the differences in the gut microbiota of C-section versus vaginally-delivered neonates begin to emerge.

Findings of bacterial DNA in the placenta, amniotic fluid, and newborn meconium have given rise to the hypothesis that the human fetus may be exposed to microbiota in utero through ingestion of amniotic fluid [16,19,20,21]. Yet no evidence exists for fetal exposure to live bacteria in healthy pregnancies. If microbiota colonize the fetal intestinal tract before labor and birth, this microbial colony should be reflected in the meconium, but not be impacted, or be impacted to a lesser extent, by delivery mode. Thus, we hypothesized that a higher delivery-mode signal should be found in the microbiota of the feces passed after meconium (i.e., transitional stool). To test this hypothesis, we compared the composition, structure and predicted metabolic function of microbiota in meconium and the subsequent transitional stool from neonates born by scheduled elective C-section vs. vaginal delivery.

## 2. Materials and Methods

### 2.1. Design and Subjects

Trained staff invited women to participate in the study if they were scheduled for a vaginal delivery or an elective C-section delivery between 38 and 42 weeks of gestation (confirmed by an ultrasound taken before the 20th week of pregnancy) in the participating private hospital in Porto Alegre, Brazil. Women were not eligible for the study if they were administered oral antibiotics in the third trimester of pregnancy; if they had diabetes, hypertensive disorders, autoimmune disorders, or HIV/AIDS during pregnancy; if they had smoked during pregnancy; or if they were on a restrictive diet during pregnancy. For vaginal births, we further only included women whose water broke less than 12 h before delivery. All vaginal deliveries were by spontaneous vaginal birth and no instruments were used. Women also had to consent to allowing study staff collect meconium or stool from their neonate, and to completing a postnatal questionnaire. The Research and Ethics Committee of the Hospital de Clinicas (protocol No. 11/0388) and the Hospital Mae de Deus (protocol No. 524/11) in Porto Alegre, Brazil, approved the study protocols and consent.

From medical records, we ascertained information on mode of delivery, gravidity, parity, history of urinary tract infection during pregnancy, antibiotic use during pregnancy, birth weight, labor length, head circumference, placenta weight, sex and race. Other clinical information was derived from a questionnaire administered after birth before discharge.

### 2.2. Sample Collection and 16S Ribosomal RNA Sequencing

Of the 89 mother-child pairs who consented and enrolled in the original study, we were able to collect 78 meconium samples (collected within 48 h of birth; all but 2 were collected <24 h). From the 78 meconium samples, 59 (75.6%) had detectable bacterial DNA. Transitional stool samples (collected 2–3 days after birth) with detectable bacterial DNA for analysis were available for 50 of 59 of these neonates. All specimens were collected from diapers with sterile spatulas, transferred to sterile tubes, which were kept at 4 °C for <6 h, and then frozen at −80 °C until DNA extraction.

Total DNA was extracted using QIAamp^®^ DNA Stool mini kit (QIAGEN) according to the manufacturer’s instructions. After DNA extraction (200 µL of final volume), we concentrated DNA samples to 20 µL using 3 M Na Acetate precipitation and kept it stored at −20 °C. We determined the DNA concentration of prepared samples using the Quant-iT PicoGreen dsDNA reagent and kit (Invitrogen, Grand Island, NY, USA) based on the manufacturer’s instructions. We amplified the V4 region of the 16S ribosomal RNA (rRNA) gene by polymerase chain reaction (PCR) using the Illumina-adapted universal primers 515F/806R [22]. PCR was done in triplicate using the Bio-Rad CFX 96 thermal cycler (Bio-Rad, Hercules, CA, USA). We then pooled the amplicons in equimolar ratios, purified them using QIA quick PCR purification kit (Qiagen Inc., Chatsworth, CA, USA), and sequenced them on the Illlumina MiSeq platform (Genome Technology Center of NYU Medical Center, NY, USA) using a paired-end technique. Reagents for DNA extraction and for PCR amplification were sequenced as negative controls.

### 2.3. 16S rRNA Gene Sequence Analysis

We analyzed 16S rRNA gene sequence data using the Quantitative Insights Into Microbial Ecology (QIIME) software package (v1.8) [23]. Using qualified sequences (Phred ≥ Q20), we identified and quantified Operational Taxonomic Units (OTUs) using an open-reference method that maps sequences with 97% identity to known sequences in the Greengenes database (v13_8) [24] using UCLUST [25] and PyNAST [26] alignment algorithms. We used ChimeraSlayer to identify chimeric sequences. Negative-control derived OTUs were discarded from the OTU table using a filtration script (filter_otus_from_otu_table.py) in QIIME.

We rarefied to 1634 reads per sample to calculate microbial diversity. We calculated alpha diversity using the phylogenetic distance and the detected number of species metrics. Principle coordinate analysis (PCoA) with weighted Unifrac distance metric were used to evaluate beta diversity (i.e., the variation in microbial community composition) between C-section and vaginally-delivered neonatal fecal samples [27]. We performed permutational multivariate analysis of variance (PERMANOVA) to determine the significance of differences in beta diversity by delivery mode. PERMANOVA, which partitions inter-group and intra-group distances, is a permutation-based extension of multivariate analysis of variance to a matrix of pairwise distances [28]. We also used non-parametric *t* tests with 10,000 Monte Carlo permutations for significant test of microbial diversity.

We used linear discriminant analysis (LDA) effect size (LEfSe) [29] to identify biologically and statistically significant differences OTU relative abundance. We then used Phylogenetics Investigation of Communities by Reconstruction of Unobserved States (PICRUSt) to predict the metabolic function of the metagenomes from the 16S *rRNA* gene dataset [30], with Kyoto Encyclopedia of Genes and Genomes (KEGG) Orthologs classification [31]. An LDA score of >3 (among OTUs with at least >1% relative abundance in any group) was used to determine significant differences in abundance of OTUs and metabolic pathways.

## 3. Results

### 3.1. Neonates and Samples

Of the 78 neonates that provided meconium samples, 59 (75.6%) had bacterial DNA present in their meconium. There were no significant differences in the clinical characteristics of mothers or neonates who had meconium with vs. without detectable bacterial DNA. Of the 59 neonates with meconium bacterial DNA, 50 also provided a subsequent transitional stool sample with bacterial DNA. We analyzed the meconium and transitional stool from these neonates for the current study. Demographic, clinical, and anthropometric characteristics of the 59 neonates (13 from vaginally-delivered neonates; 46 from C-section neonates) who had meconium with detectable bacterial DNA (and their mothers) are presented in Table 1 according to delivery mode.

### 3.2. Microbial Community Diversity, Abundance and Predicted Function

**Figure 1:**
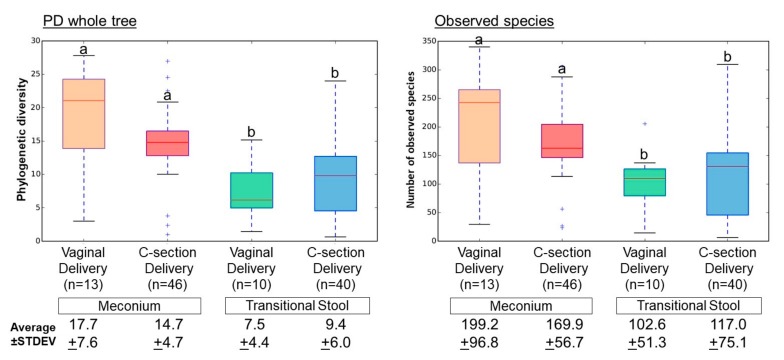
Bacterial alpha diversity in the meconium and transitional stool by delivery mode. Samples were rarefied to 1634 reads per sample. The nonparametric *p* values were calculated using 100,000 Monte Carlo permutation. Different letters indicate significant differences (e.g., ‘a’ is significantly different from ‘b’, but not significantly different from ‘a’); Statistical significance for phylogenetic diversity (PD) whole tree, *p* < 0.005; Observed species, *p* < 0.05. +, outlier samples.

We obtained 604,570 sequences (paired-end, Phred ≥ Q20) from the 59 meconium samples, and 472,165 sequences from the 50 transitional stools. There was an average of 9878 reads per sample, binned into 5939 Operating Taxonomic Units (OTUs; Table S1). Alpha diversity was greater for meconium than for transitional stool samples, regardless of delivery mode (Figure 1; Phylogenetic diversity whole tree, *p* < 0.005; Observed species, *p* < 0.05).

**Figure 2:**
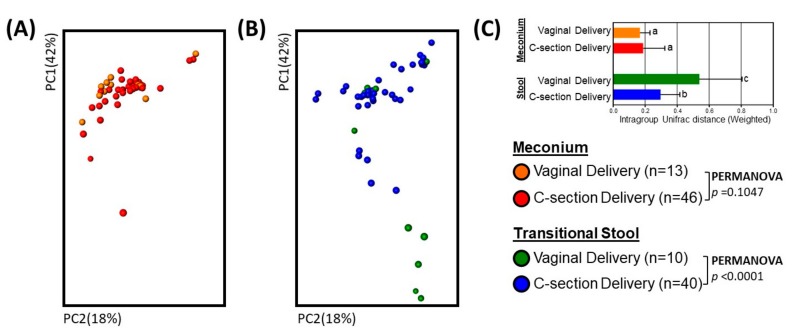
Bacterial diversity in the meconium and transitional stool by delivery mode. (**A**–**C**) Principal Coordinate Analysis (PCoA) plot of bacterial communities in meconium (**A**) and transitional stool (**B**) by delivery mode. Weighted UniFrac distances were used to evaluate diversity between samples. PERMANOVA was used to test dissimilarity. (**C**) Box plot of intra-group distances. The non-parametric *p* values were calculated using 100,000 Monte Carlo permutation. Different letters (a, b and c) indicate significant differences; *p* < 0.001.

Microbial community structure (beta diversity) did not differ significantly by delivery mode in the meconium (Figure 2A; Weighted UniFrac; PERMANOVA, *p* = 0.1047). It did, however, vary in the transitional stools (Figure 2B; Weighted UniFrac; PERMANOVA, *p* < 0.0001). There were also inter-individual differences in microbiota (Figure S1), which varied by group. Transitional stool samples had higher variability then meconium, particularly transitional stools from babies born vaginally (Figure 2D and Figures S1 and S2A; Weighted UniFrac; non-parametric *t*-test, *p* < 0.001).

**Figure 3:**
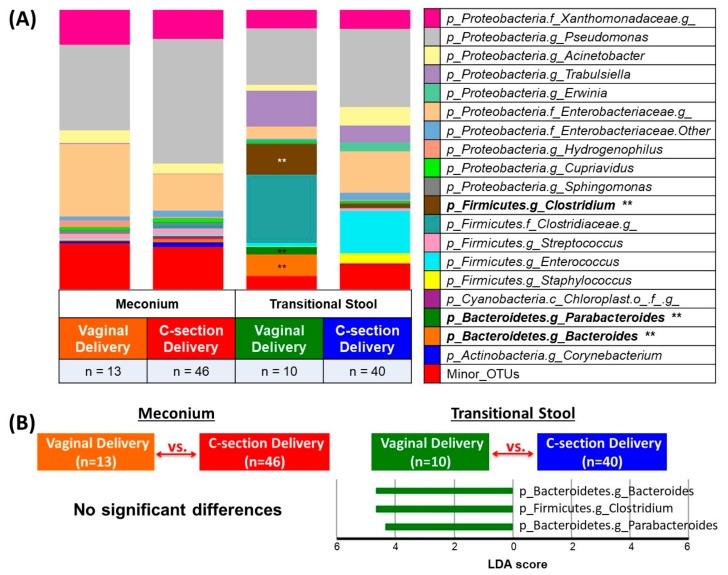
Bacterial taxa comparisons in meconium and transitional stool by delivery mode. (**A**) Each taxonomy (>1% of average relative abundance in any groups) is indicated by a different color at the genus level. ** Indicates overrepresented taxa (using LDA > 3.0) in comparisons of delivery mode within sample type. (**B**) Histogram of overrepresented taxa (using LDA > 3.0) in each group.

Both variability and the bacterial community structure in the neonatal gut changed during the transition from meconium to the transitional stool (Figure 2A,B and Figures S1 and S2A). We used linear discriminant analysis (LDA) effect size (LEfSe), retaining bacterial OTUs with >1% abundance, and found no significant delivery-mode differences in meconium microbiota. However, in the transitional-stool microbiota, LEfSe detected a significantly higher relative abundance of the genera *Bacteroidetes*, *Parabacteroides* and *Clostridium* in vaginally-delivered neonates as compared to C-section delivered neonates (LDA > 3.0; Figure 3).

Microbial metagenomic functioning predictions using PICRUSt provided further insight into the predicted metagenome functional profiles, again showing delivery-mode differences in the transitional stools, but not in the meconium samples (Table 2). Of note, transitional stool from the vaginally-delivered neonates had a higher proportion of bacterial genes related to amino sugar and nucleotide sugar metabolism, whereas stool microbiota from C-section neonates had higher abundance of genes related to tryptophan, valine, leucine and isoleucine degradation, fatty acid metabolism, and xenobiotics biodegradation and metabolism (LDA > 3.0; Table 2). Interestingly, microbiota differences in the transition of C-section neonatal fecal samples (Figure S2A) did not translate into differences in predicted microbial-metagenomic functional profiles, as they did in vaginally-delivered neonatal fecal samples (Table 2; Figure S2B). These results indicate relatively slower maturation of microbiome metabolic functions in the gut of C-section vs. vaginally-delivered neonates during the first days of life.

## 4. Discussion

In our cohort from Southern Brazil, we found that the transition from meconium to stool involves altered bacterial structure, composition and decreasing alpha diversity, and that delivery mode-associated differences in bacterial diversity, OTU abundances and predicted metagenomic functions arise not in the meconium, but in the transitional stool passed after meconium.

Our findings on the transition in microbiome composition and structure from meconium to transitional stool are consistent with previous work reporting loss of alpha diversity postnatally in mice [32] and human neonates [33]. Selective environmental pressures, such as human milk oligosaccharides found in breast milk, may drive the reduction in microbial diversity from meconium to transitional stool. Our finding (Figure S2A) that initial blooms of *Streptococous* and several Proteobacteria phylotypes in meconium were replaced by bacteria in the *Clostridiaceae* family (e.g., *Lactobacillus* spp.) in transitional stool of vaginally born neonates, is also consistent with findings in mice [32]. *Lactobacilli* are facultative anaerobes that ferment milk lactose and casein and produce lactate [34,35,36]. Lactic acid lowers the pH of the intestinal contents, inhibiting growth of obligate anaerobes [35,37] commonly found in meconium. The role of this initial reduction in diversity on education of host immune development and metabolic health remains to be elucidated.

Previous investigations on the impact of delivery mode on meconium bacterial composition have been mixed, with some studies having found differences [15,16,38], and others [39] not. Rocio et al. reported differences in bacterial counts in the meconium by mode of delivery among 108 neonates (80 vaginally-delivered and 28 C-section) [16]. They also reported differences in microbiota composition over the first 3 months, analyzing 33 different bacterial taxa by quantitative reverse transcription PCR in meconium, 2nd-day stool (transitional stool), and 7-, 30-, and 90-day stools; it is unclear from their study when compositional differences emerged [16]. Our statistical power to detect large differences may have been limited by the relatively small number of vaginally-delivered neonates in our sample. However, we were able to discern microbiota differences in transitional stools from 10 vaginally-delivered and 40 C-section neonates, and these findings are consistent with a larger sample of transitional stools [40]. Discrepancies between our study and other studies on this topic might reflect collection, storage processing or analytic platforms [41]. Differences may also be due to inter-hospital differences in delivery-mode practices, including intrapartum antibiotic administration. While we excluded 3rd-trimester antibiotics, the standard protocol for operative C-section in our hospital in Porto Alegre is intravenous intrapartum administration of cefazolin (half-life ~120 min), 30 to 60 min before surgery [42,43]. A recent study found intrapartum antibiotics were associated with an impacted infant microbiome [14]. Thus, it is possible the impact of C-section observed in our study includes the effect of intrapartum antibiotics.

Our amplicon-sequencing results compliment previous studies [10,11,12] that demonstrated mother-to-newborn transmission at the strain level. Makino et al. have shown that stains found in the mother before delivery, were also found in the meconium [10]. They have also reported the differences in transmission *Bifidobacterium* strains by delivery mode in 12 vaginally-delivered neonates and 5 C-section neonates, but only at 3 days after birth [11]. In the light of studies showing evidence of bacterial DNA in amniotic fluid [44,45], chorioamnion tissue [46], cord blood [47], fetal membranes [48], and the placenta [19], some investigators have taken evidence of bacterial DNA in meconium to suggest potential translocation of microbiota from mother to fetus before birth. This has been called the ‘in-utero colonization hypothesis’ [41]. However, no study, to our knowledge, has shown evidence of live bacteria in utero in healthy pregnancies. Regardless of viability, intrauterine maternal bacterial DNA might be important in intestinal transcriptional profiles programming of the offspring conferring to them protection against pathogen colonization [49].

The detection of bacterial DNA in the meconium samples in our study does not clearly support nor refute the in-utero colonization hypothesis, as meconium may reflect not only prenatal but also peri- and postnatal exposures [41]. About 75% of meconium samples in our study contained detectable microbial DNA; other studies have found similar [16] or lower prevalence of microbes in meconium [50,51,52]. Presence of microbes in meconium has been shown to increase with time [51], which one might take to support the hypothesis that intestinal colonization starts postnatally [41]. Yet, if the bacterial DNA present in the meconium of our study was acquired postnatally, one would expect the composition and structure to be altered by delivery mode, as was observed in the microbiota of the subsequent transitional stools in our study.

## 5. Conclusions

In summary, we found evidence of bacterial DNA in most meconium samples and we observed that bacterial diversity in the neonatal intestinal tract decreases in the first days of life, from the passing of meconium to transitional stool. Moreover, the initial transition in neonatal intestinal bacterial structure, composition and predicted metagenome function appears to be impacted by delivery mode. Our findings also add to the literature that vaginal birth-acquired microbes are important in the normal development of the early neonatal gut microbiota development and also their metabolic function, since C-section delivered neonates fail to show transitional changes in predicted bacterial metagenomic functions. Future research should determine whether the differential transition of these pioneering microbial communities and their metagenomic functions are on the pathway to the metabolic and immune-mediated diseases that have been associated with C-section delivery.

## Figures and Tables

**Table 1:** Characteristics * of Brazilian mothers and their newborns who had meconium microbiota.

	Vaginal Delivery (*n* = 13)	C-Section Delivery (*n* = 46)
*Maternal characteristics*		
Mothers age, y	28.5 (5.9)	30.5 (4.7)
Mothers race		
White, *n* (%)	12 (92)	42 (91)
Non-white, *n* (%)	1 (8)	4 (9)
Urinary tract infection		
Yes, *n* (%)	4 (31)	6 (13)
No, *n* (%)	9 (69)	40 (87)
Medications in pregnancy		
Yes, *n* (%)	4 (31)	19 (41)
No, *n* (%)	9 (69)	27 (59)
Antibiotics in pregnancy		
None, *n* (%)	9 (69)	36 (78)
1st Trimester, *n* (%)	1 (8)	5 (11)
2nd Trimester, *n* (%)	3 (23)	5 (11)
Ever smoke tobacco		
Yes (%)	0 (0)	12 (26)
No (%)	13 (100)	34 (74)
Pre-pregnancy BMI, kg/m^2^	22.4 (3.2)	25.3 (5.0)
Pregnancy weight gain, kg	13.0 (3.0)	13.7 (5.4)
*Newborn characteristics*		
Sex		
Boy, *n* (%)	3 (23)	30 (65)
Girl, *n* (%)	10 (77)	16 (35)
Birth weight, g	3127.6 (334.5)	3234.3 (427.4)
Birth length, cm	48.7 (2.2)	48.9 (1.6)
Head circumference, cm ^†^	35.9 (4.4)	35.7 (2.8)
Placenta weight, g ^§^	674.8 (91.3)	624.2 (131.7)
Breastfed 1st 24 h		
Yes, *n* (%)	13 (100)	40 (87)
No, *n* (%)	0 (0)	6 (13)

* values expressed as mean (standard deviation) if not otherwise indicated as *n* (%); ^†^ based on 10 vaginally-delivered and 43 C-section delivered neonates with data; ^§^ based on 10 vaginally-delivered and 39 C-section delivered neonates with data.

**Table 2:** Predictive KEGG functional profiling of microbiota from transitional stools—but not from meconium—differ by delivery mode. *

		Transitional Stool			Meconium			
KEGG Functional Categories		Vaginal Delivery (*n* = 10)	Vs. ⇔	C-Section Delivery (*n* = 40)	Vaginal Delivery (*n* = 13)	Vs. ⇔	C-Section Delivery (*n* = 46)
Level 2	Level 3	LDA	*P* Value	LDA			
Amino Acid Metabolism	Tryptophan metabolism	-	0.01635	→ 3.06	No significant differences		
Amino Acid Metabolism	Valine/leucine/isoleucine degradation	-	0.0066	→ 3.12			
Carbohydrate Metabolism	Amino sugar/nucleotide sugar metabolism	3.16 ←	0.03282	-			
Enzyme Families	Peptidases	3.20 ←	0.0049	-			
Lipid Metabolism	Fatty acid metabolism	-	0.00946	→ 3.04			
Metabolism of Cofactors/Vitamins	-	3.22 ←	0.03282	-			
Metabolism of Cofactors/Vitamins	Porphyrin/chlorophyll metabolism	3.03 ←	0.02905	-			
Metabolism of Terpenoids/Polyketides	-	-	0.01864	→ 3.01			
Xenobiotics Biodegradation and Metabolism	-	-	0.00881	→ 3.67

* Significantly overrepresented KEGG functional categories in predicted metagenomes by delivery mode were detected using Linear Discriminant Analysis (LDA) Effect Size >3.0-fold with direction indicated by arrow (← or →).

## Supplementary Materials

The following are available online at: www.mdpi.com/2073-4425/8/12/364/s1. Correspondence and requests for materials should be addressed to N.T.M. and M.G.D.-B.
