# Supplementary material for: Delivery Mode and the Transition of Pioneering Gut-Microbiota Structure, Composition and Predicted Metabolic Function
Source: Genes (Basel). 2017 Dec 4;8(12):364. doi: 10.3390/genes8120364 (PMC5748682; doi:10.3390/genes8120364)
Supplement: Supplementary file 1 [file genes-08-00364-s001.pdf]

Table S1. Summary of analyzed sequence information.

	Vaginal Delivered Neonates		C-section Delivered Neonates		Total
**Sample type**	**Meconium**	**Stool**	**Meconium**	**Stool**	
**Number of samples**	13	10	46	40	109
**Number of sequences**	130,990	106,891	473,580	365,274	1,076,735
**Average sequences ± SD**	10,076 ±3,642	10,689 ±4,057	10,295 ±4,128	9,132 ±4,146	9,878 ±4,115
**Number of sequences yielding OTUs**	99,157	83,027	467,875	269,314	827,044
**Average sequences yielding OTUs ± SD**	7,627 ±3,426	8,303 ±3,388	8,164 ±3,420	6,733 ±3,931	7,588 ±3,676
**Number of OTUs**	2,215	1,224	3,871	2,926	5,939
**Average OTUs ± SD**	373 ±169	205 ±94	346 ±133	218 ±148	289 ±156

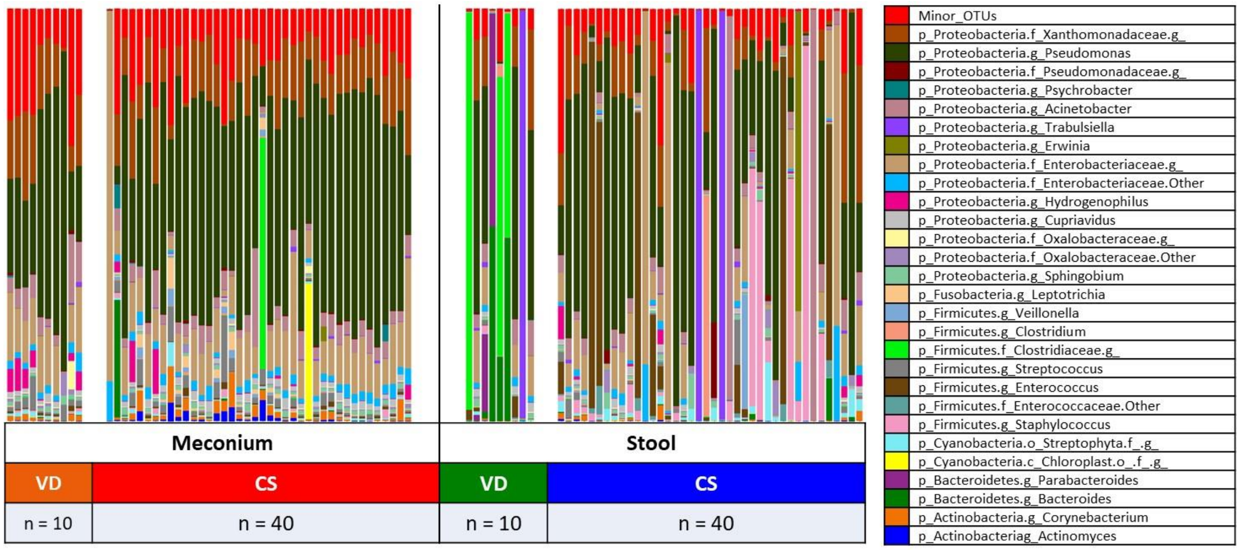

Figure S1. Differences in relative abundance of bacterial taxonomy in meconium or transitional stools according to mode of delivery. Bacterial taxonomic plots are shown at the lowest identifiable level, indicated by the letter preceding the underscore; f, family; g, genus. Each taxonomy (>1% of average relative abundance in any groups) is indicated by a different color at the genus level.

(A)

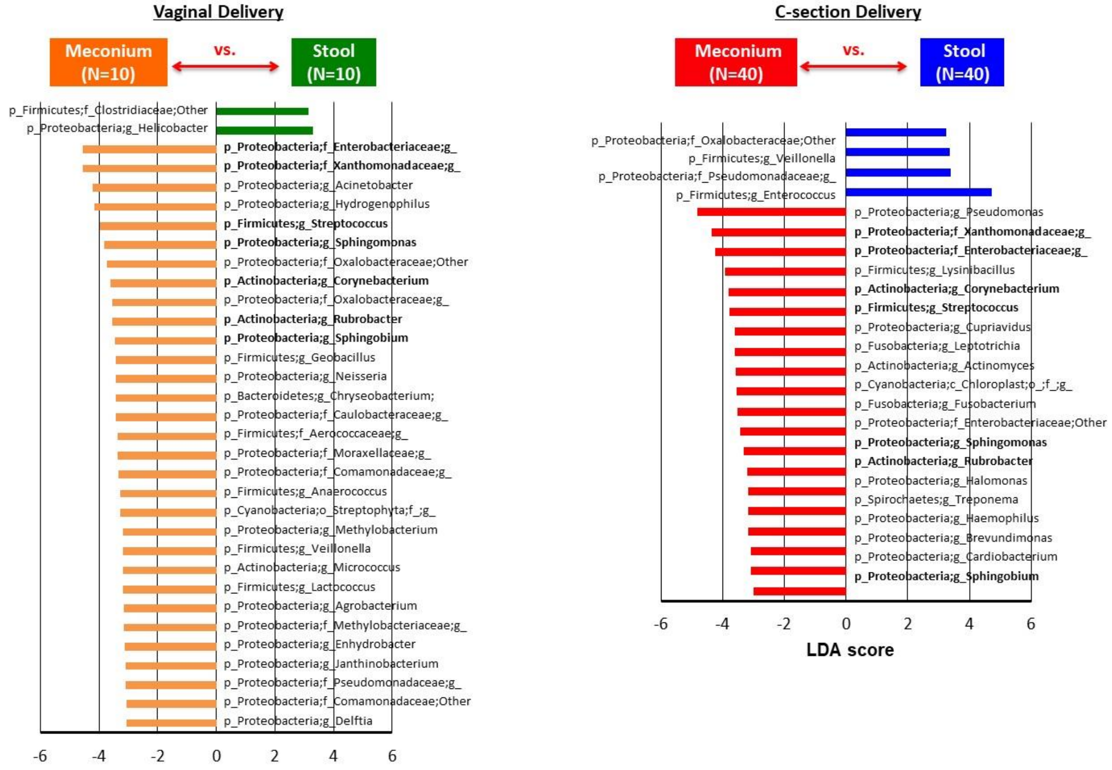

(B)

KO Functional Categories

Level 2	Level 3	Vaginal Delivery: Meconium (n=10) P value	LDA	Vaginal Delivery: Stool (n=10) P value	C-section: Meconium (n=40) P value	LDA	C-section: Stool (n=40) P value
Amino Acid Metabolism	Valine_leucine and isoleucine degradation	0.04125	3.03		-	-	-
Xenobiotics Biodegradation and Metabolism		0.02334	3.47		-	-	-
Carbohydrate Metabolism	Amino sugar and nucleotide sugar metabolism		3.10	0.03429	-	-	-
Enzyme Families	Peptidases		3.07	0.04125	-	-	-
Carbohydrate Metabolism	Fructose and mannose metabolism		3.04	0.02837	-	-	-

Figure S2. Bacterial taxa **(A)** and Predictive functional profiling **(B)** comparisons between the meconium and transitional stool according to mode of delivery. **(A)** Histogram of overrepresented taxa in each group (LDA > 3.0). Bacterial taxa detected in both of delivery mode indicates using bold. **(B)** Overrepresented metabolic routes in predicted metagenomes were detected using LDA Effect Size (>3.0-fold) test.
